# Expanding Awareness of Docosahexaenoic Acid during Pregnancy

**DOI:** 10.3390/nu5041098

**Published:** 2013-04-02

**Authors:** Rebecca Emmett, Shann Akkersdyk, Heather Yeatman, Barbara J. Meyer

**Affiliations:** 1 School of Health Sciences, University of Wollongong, Northfields Avenue, Wollongong, NSW 2522, Australia; E-Mails: rd552@uowmail.edu.au (R.E.); shannakk@yahoo.co.uk (S.A.); hyeatman@uow.edu.au (H.Y.); 2 Metabolic Research Centre, University of Wollongong, Northfields Avenue, Wollongong, NSW 2522, Australia

**Keywords:** docosahexaenoic acid, omega-3, pregnancy, fish, education

## Abstract

Pregnant women do not currently meet the consensus recommendation for docosahexaenoic acid (DHA) (≥200 mg/day). Pregnant women in Australia are not receiving information on the importance of DHA during pregnancy. DHA pregnancy education materials were developed using current scientific literature, and tested for readability and design aesthetics. The study aimed to evaluate their usefulness, the desire for pregnant women to receive these materials and whether a larger separate study (using a control group) is warranted to evaluate the influence the materials may have on increasing DHA consumption in pregnant women in Australia. Pregnant women (*N* = 118) were recruited at antenatal clinics at two NSW hospitals. Participants completed a 16-item questionnaire and DHA educational materials (pamphlet and shopping card) were provided. Participants were contacted via phone two weeks later and completed the second questionnaire (25-item, *N* = 74). Statistics were conducted in SPSS and qualitative data were analysed to identify common themes. Ninety three percent of women found the materials useful, with the main reason being it expanded their knowledge of DHA food sources. Only 34% of women had received prior information on DHA, yet 68% said they would like to receive information. Due to the small sample size and lack of a control group, this small study cannot provide a cause and effect relationship between the materials and nutrition related behaviours or knowledge, however the results indicate a potential positive influence towards increased fish consumption and awareness of DHA containing foods. This suggests a larger study, with a control group is warranted to identify the impact such materials could have on Australian pregnant women.

## 1. Introduction

Docosahexaenoic acid (DHA) is a long chain omega-3 polyunsaturated fatty acid (LC *n-*3 PUFA) essential for neural, visual and cognitive development of the growing fetus [1,2]. For this reason, DHA consumption during pregnancy is important and women should be supplied with adequate DHA education during gestation [3]. There are many nutrients of particular importance during pregnancy, yet the main focus appears to be predominately on folate, iron and calcium [3]. The importance and health benefits of DHA are rarely communicated to pregnant women in Australia [3] and they have been found to lack knowledge of DHA and consume suboptimal levels during pregnancy [3,4].

We have shown that the median intake of total LC *n-*3 PUFA in the Australian female population is 120 mg per day [5]. The consensus statement for dietary fats and the International Society for the Study of Fatty Acids and Lipids (ISSFAL) have developed specific recommendations for DHA alone, using scientific evidence that is based on the physiological effects of DHA, and they suggest pregnant women consume at least 200 mg/300 mg (respectively) of DHA per day [6,7]. The Omega-3 Centre of Australia and New Zealand also recommend 200 mg DHA per day during pregnancy and lactation [8]. In the third trimester of pregnancy the fetus alone accumulates 67 mg of DHA per day [9]. Several studies suggest inadequate DHA intake is associated with decreased maternal DHA status, as the fetus takes what it needs from maternal stores [2,10]. Pregnant women in Australia are only consuming an average of 99 mg DHA/day [4]. This falls short of the 200–300 mg DHA per day as recommended by the European consensus, ISSFAL and the Omega-3 Centre [6,7,8].

Very few pregnancy education materials exist on LC n-3 PUFAs, and of those that do, the focus is on safe fish consumption [3,11]. Food Standards Australia and New Zealand have an information card which provides advice on healthy fish consumption for pregnancy, but does not refer specifically to DHA or other food sources of LC *n*-3 PUFAs [11]. There are currently no pregnancy education materials available in Australia which provide information on the health benefits and importance of DHA for pregnancy or DHA food sources other than fish [3]. Butler *et al.* [12] identified that pregnant women feel they are only given information on what foods to avoid, however they would also like to know what foods to eat to obtain optimal health benefits for their babies. Therefore, information on the health benefits, importance and sources of DHA during pregnancy is warranted.

In considering the development of a communication tool that delivers scientific nutritional knowledge, the focus should be how to translate the message into a form that can be easily read and understood by the general population with varying education levels [13]. There are multiple ways to communicate important information, namely television advertising, websites, interactive computer games and more traditional print media. Research carried out in the area of delivering complex nutritional information, such as a recommended fat intake, has found that using a one page handout greatly improved participants’ understanding of different types of fats [14]. Pamphlets have also been identified as a preferred channel for receiving information and pregnant women want practical information in language they can understand [3].

The aims of this study were to: (i) design DHA educational materials in the form of a pamphlet and wallet sized shopping card; (ii) assess the appropriateness of the design and the ability of the materials to deliver a clear message; (iii) assess women’s desire to receive the materials during pregnancy; and (iv) evaluate the usefulness of the materials to identify whether a larger study involving a control group should be conducted to indentify the impact the materials may have on increasing DHA knowledge and consumption.

## 2. Methods

Ethics approval was granted by the University of Wollongong/South Eastern and Illawarra Area Health Service Human Research Ethics Committee in Australia. The ethics reference number is HE10/193 and approval was granted on the 04/08/2010.

### 2.1. Development and Pilot Testing of Education Materials

A pamphlet and wallet sized shopping card were developed based on the most up to date scientific literature on DHA during pregnancy [6,7,8] and professional advice from academic experts and professional societies in the field of fatty acids (Omega-3 Centre and ISSFAL). The materials were designed to educate women about the importance of DHA during pregnancy and breastfeeding and to provide practical advice on how to achieve the European consensus recommendations of at least 200 mg DHA/day [6,7]. The practical advice component of the materials was developed with the aim to reduce barriers to change by providing simple solutions for achieving at least 200 mg DHA/day. The pamphlet provided suggested meals and each meal was rated using varying numbers of stars (*i.e.*, ∗) according to how much DHA is present in the meal (*i.e.*, if the meal contained 50 mg DHA, it received one star (∗) and if the meal contained 200 mg DHA it received 4 stars (∗∗∗∗)). The pamphlet gives instructions for the participants to consume 4 stars a day to meet the recommended 200 mg DHA per day. Food safety issues, nutritional adequacy and easy to understand terminology were carefully considered in all aspects of the design process, so as to ensure the materials were clear, precise and in line with current nutritional guidelines. Permission was obtained from Wollongong University and Omega-3 Centre for use of their logos. 

A small cross-sectional study of adult female participants (*N* = 15, convenience sample: recruited by approaching women from the public) was conducted to test design and content of the materials. Women were given the materials to read and then asked to complete a six-item survey, with quantitative and qualitative questions about the design (colour, text size, readability), main message portrayed, amount of information and ease of understanding of the information in the materials. Responses from each questionnaire were entered into an excel spreadsheet (Excel for Microsoft Windows 2003, software, Version 11, Microsoft Corporation, United States) and the overall outcomes were analysed qualitatively by using key words to identify common themes and quantitatively by measuring closed question responses. Outcomes were used to make seven changes to the materials to improve the design and understanding.

### 2.2. Evaluation of Education Materials

Two surveys were developed for use in the evaluation study. Survey one was designed to determine the participants’ current DHA consumption behaviours during pregnancy (fish intake and shopping for DHA enriched products), if they had received any DHA information during their pregnancy and whether they would like to receive DHA information during pregnancy. The self-administered survey was separated into two sections, a 10-item DHA section and a six-item demographic section. Survey two was a 25-item questionnaire designed to assess the participants’ perceived usefulness of the materials, changes in opinion since receiving the materials, preferred time and place to receive these materials and suggestions to make the materials more useful.

Pregnant women were recruited at antenatal sessions during September 2010 in two public hospitals—one in Sydney and one in the Illawarra region of New South Wales, using information sheets and flyers. Participants were required to sign a consent form for both surveys and provide follow up details for future contact. Researchers were available for assistance with completion of the surveys if required. Exclusion criteria were <18 years of age, not pregnant or spoke no English. Upon completion of the survey and consent form, the participants received the pamphlet and shopping card. Two weeks after the first survey was administered the researchers conducted the second survey over the phone. At least three attempts were made over a period of a week to call each participant.

Data from each survey were entered into Statistical Package for Social Sciences (SPSS) (SPSS Version 17.0: 2006, SPSS Inc., Chicago, IL, USA) and coded for analysis. Qualitative responses were grouped into themes to assess common ideas and views. Quantitative data were tested for normality using Shapiro-Wilk tests. Means, standard deviations (SD), frequencies and percentages were carried out on all quantitative questions. A score was assigned to each demographic variable to allow means and standard deviations to be calculated, for example: Single = 1, Married = 2, De facto = 3 (where De facto means living with partner but not married to partner). Wilcoxon Signed Ranks Tests were used to assess changes in responses between the first and second survey. Chi squared tests were used to assess statistically significant relationships between demographic variables and responses. A level of significance of 0.05 was used for all analyses.

## 3. Results

### 3.1. Recruitment/Demographics

A total of 144 pregnant women were invited to participate in the evaluation study by completing the first survey, of which 118 (82%) agreed. The second survey had a response rate of 63% (*N* = 74). Of the 44 women who did not complete the second survey, 28 did not answer their phone, five said they no longer wanted to be in the study either because they had just had their baby and were too busy, were in labour or just did not want to do the second survey, four phone numbers were disconnected, two women did not leave a return phone number, one phone number was a wrong number, two women asked to be emailed but did not reply to the email and two women had their phones turned off. Participant characteristics are shown in Table 1.

**Table 1:** Characteristics of participants in initial recruitment and follow up recruitment.

Characteristic		Initial recruitment *		Follow up recruitment ^†^		Significance
		(118 participants)		(74 participants)		
		*n*	%	*n*	%	*p* value
Average weeks pregnant		30.2		29.4		0.4
SD		7.5		7.2		
Trimester						
	First (1–13 weeks)	7	5.9	4	5.7	
	Second (14–26 weeks)	24	20.3	20	27	
	Third (27–41 weeks)	87	73.7	50	67.6	
Mean		2.7		2.6		0.5
SD		0.6		0.6		
Parity						
	0	84	71.2	54	73	
	1	23	19.5	15	20.3	
	2	9	7.6	5	6.8	
	3	1	0.8	0	0	
	≥4	1	0.8	0	0	
Mean		0.41		0.3		0.5
SD		0.74		0.6		
Marital status						
	Single	6	5.1	3	4.1	
	Married	82	69.5	55	74.3	
	De facto	30	25.4	16	21.6	
Mean		2.2		2.2		0.7
SD		0.5		0.5		
Household income						
	Unanswered	7	5.9	2	2.7	
	Below $10 K	9	7.6	4	5.4	
	Between $10 and $20 K	2	1.7	2	2.7	
	Between $20 and $40 K	10	8.5	8	10.8	
	Between $40 and $75 K	32	27.1	16	21.6	
	Over $75 K	58	49.2	42	56.8	
Mean		4.0		4.2		0.4
SD		1.3		1.2		
Highest level of education						
	Unanswered	1	0.8	0	0	
	Primary	0	0	0	0	
	Secondary	20	16.9	9	12.2	
	TAFE	29	24.6	18	24.3	
	College	3	2.5	3	4.1	
	University UG	28	23.7	19	25.7	
	University PG	37	31.4	25	33.8	
Mean		4.2		4.4		0.4
SD		1.6		1.5		

De facto: living with partner but not married to partner, TAFE: Training and Further Education Courses, UG: undergraduate, PG: postgraduate. * Completed survey 1, ^†^ Completed both survey 1 and survey 2.

### 3.2. Survey 1 Outcomes (*N* = 118)

When asked how often fish and/or seafood was consumed during pregnancy, the most common consumption reported was once per fortnight (23%), followed closely by once per week (22%) and twice per week (19%). Education level was significantly related to fish consumption: women who had a tertiary education were more likely to report higher fish consumption than women of secondary only education (*X*^2^ 4.821, *p* = 0.028). Only 34% of all women reported they had received information from their health care professional on DHA during pregnancy, whereas 68% said they would like to receive information on DHA during pregnancy.

### 3.3. Survey 2 Outcomes (*N* = 74)

The sample (*N* = 74) of women who completed both surveys had similar characteristics compared with the initial recruitment sample (*N* = 118), as there were no significant differences between the group characteristics (Table 1). It is conceivable that these 74 participants were more interested in the potential benefits of DHA than those participants that did not respond a second time. These participants (*N* = 74) showed an increase in reported fish consumption from an average of 5 times per month (±SD 4.5) in the first survey to an average of 6.6 times per month (±SD 4.5) in the second survey (*p* < 0.001). When participants who had (previously) received DHA information from their health care professional (*N* = 10) were excluded from the analysis, the increase was still statistically significant (*p* < 0.001). Similarly there was a significant increase in the number of women who reported actively shopping for DHA enriched food products after receiving the DHA material (7/74 to 28/74; *p* < 0.001). After receiving the materials, the number of women who answered yes to wanting DHA information (like the pamphlet and card) for pregnancy, increased from 55 (74%) to 70 (95%) (*p* < 0.001). The awareness of DHA food sources also significantly increased, as there was an increase in the number of times participants reported seafood, eggs, enriched milk, enriched bread and meats as DHA food sources as shown in Table 2. When asked what foods participants knew to contain DHA a small number (4) of women listed supplements and/or fish oil. Out of the 74 women who completed both surveys 1 woman listed fish oil/supplements as a source of DHA in the first survey and 3 women listed fish oil/supplements as a source of DHA in the second survey, this was a non-significant increase (*p* > 0.05).

**Table 2:** Change in number of times foods were listed as containing DHA before compared to after receiving the materials.

Food	Survey 1 (*n* = 74)	Survey 2 (*n* = 74)	Change	*p* Value *
Fish	46	56	10	0.068
Seafood	5	13	8	0.045
Eggs	16	29	13	0.011
Milk (enriched)	12	22	10	0.007
Yoghurt (enriched)	5	8	3	0.369
Bread (enriched)	10	22	12	0.002
Meats	0	4	4	0.045
Average amount of DHA foods listed per participant	1.3	2.1	0.8	<0.001
SD	0.9	1.3		

DHA: Docosahexaenoic Acid (* Wilcoxon Nonparametric Test).

Most women reported using (includes reading) the pamphlet and/or card, 59/74 and 48/74 respectively. The card was carried in their wallet by 43/74 of women. Almost all women (72/74) wanted to keep the materials for the duration of their pregnancy. Almost half of women (34/74) said the materials changed their eating habits in some way. Women who answered yes to the materials changing their eating habits were asked to provide responses of a qualitative nature as to how they believed their eating habits had changed. The responses were grouped into common themes and four main themes were identified: women perceived an improvement in their diet, women perceived an increase in their fish intake, women perceived they had an increase of DHA *enriched* foods in their diet and women perceived an increase in DHA foods in general in their diet (not exclusive to enriched). Some of the comments which resulted in these themes are listed below:

“Increased healthy eating”,

“Eating more fish”,

“More conscious effort of making sure I do eat fish and omega-3 enriched bread now”,

“Try to eat more fish now and have started taking capsules”,

“Eating more bread that has omega-3 and actively looking for omega-3 products”.

Of the 34 women who said they changed their eating habits, their answers to the quantitative questions in the survey showed: 19 reported an increase in fish consumption between the first and second questionnaire, 16 said “yes” they now actively shopped for DHA enriched foods whereas “no” they did not before receiving the materials and 9 reported both an increase in fish consumption and actively shopping for DHA enriched foods. There was a statistically significant relationship between reported/perceived changes in eating habits and shopping for DHA enriched foods (*X*^2^ 8.409, *p* = 0.004).

**Figure 1:**
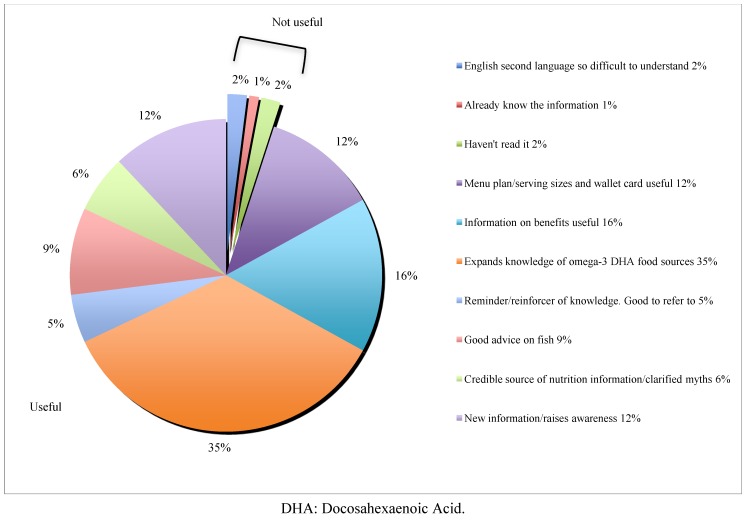
Reasons women found the DHA pregnancy education pamphlet and card useful or not useful. The reasons reported were of qualitative nature and have been grouped into common themes; these themes are displayed in the figure.

In total 69/74 of women who completed both surveys said they found the information useful and 5/74 did not. Women found the information in the materials useful or not useful for a variety of different reasons, which were reported by women in a qualitative nature and grouped into common themes by the researchers (Figure 1). The two main reasons for usefulness were that the materials expanded knowledge of DHA food sources and there was information on the benefits of DHA. A minority of the women found the information not useful due to difficulties understanding the written English and not having the time to read the information (Figure 1).

## 4. Discussion

The present study identified that women found provision of DHA pregnancy education materials to be useful in providing information on the health benefits of DHA and after provision of the materials women had increased awareness of DHA food sources. Behaviour changes, including increased fish consumption and actively shopping for DHA food sources, were reported to have occurred. However, a control group and the inclusion of food intake records such as a food frequency questionnaire or food diary would be required to identify whether these reported changes in behaviour did actually occur and to what extent. A desire to receive the materials existed among the target population. Evaluation of the results suggests the materials designed in the present study are useful nutrition education tools, which may have potential to assist in increased consumption of DHA during pregnancy.

In terms of the aesthetics and message delivery, the results indicate the design of the materials was well accepted and the materials delivered a clear and main message: “DHA and fish are important during pregnancy”. Clear delivery of an education message may lead to increased awareness and more successful behaviour modification [15,16]. These findings support other research, which suggests there is a strong importance for a clear message in education materials [16].

The materials developed in the present study were designed to educate women on the importance of DHA during pregnancy (benefits) and to provide practical advice on how to achieve a desirable amount of DHA, which are principles consistent with the Health Behaviour Model [17]. A portion of women in the study highlighted the practical aspects of the materials as a reason for finding the materials useful. This finding suggests education materials with a practical aspect may have increased usefulness compared to those without. The Health Behavioural Model suggests ‘cues to action’ may influence behaviour change [17]. The wallet sized shopping card provided to the participants may have acted as a cue for alternative food choices when shopping. Further research should investigate the significance of the practical suggestions and tools in DHA education materials versus simply providing information on the topic.

Raised awareness of DHA food sources may be linked with the reported increase in fish consumption and actively shopping for DHA foods in the present study. Increased awareness and knowledge has been shown to positively impact on behaviour adoption [15,16,18]. Torvaldsen *et al.* [18] found provision of a Listeria education pamphlet increased the likelihood of women identifying foods which should be avoided during pregnancy. Results from a recent cross-sectional study of 190 pregnant women indicate that women are likely to change their eating behaviours when provided with appropriate information [3]. Future research should determine the degree to which the DHA education materials may be associated with self-reported behaviour changes.

The results of the present study indicate that prior to receiving information on DHA during pregnancy, more highly educated women are consuming greater amounts of fish/seafood during pregnancy compared with less educated women. Sinikovic *et al.* [3] found that women with higher levels of education are more likely to seek information on LC *n*-3 PUFAs during pregnancy. These findings suggest that women of higher education level may be more aware of the benefits of DHA and are therefore consuming more fish/seafood during pregnancy compared to other women [3]. As such, there may be a specific need for DHA pregnancy education to target women of lower education levels. 

Education is an important aspect of pregnancy care, yet prior studies have found that women expect to receive more nutrition information from their doctor, midwife or health care professional than they actually receive [19]. The present study identified that only 34% of women had received any information on DHA from their healthcare professional. Likewise Sinikovic *et al.* [3] identified that only 23% of pregnant women had received any LC *n*-3 PUFA information from their healthcare professional. This indicates that no more than one third of women are receiving education on DHA during pregnancy [3]. 

It is concerning that the benefits of DHA are not being communicated to pregnant women, especially considering prior studies have identified a high interest in nutrition information by women during pregnancy [12,19]. There are available relevant education materials for pregnant women on including fish in their diet [11], however the materials focus on avoiding certain types of fish known to be high in mercury, rather than highlighting the benefits of consuming fish for its DHA content. Such a focus on a negative attribute of food (mercury) may lead to women avoiding consumption of fish during pregnancy. This may occur when different agencies publish different advice (for example the WHO recommends avoiding tuna [20] while FSANZ does not [11]), leaving pregnant women unsure of the risks associated particular fish species and reducing their fish consumption [21]. A desire for women to receive DHA information during pregnancy was reported by women in the present study and the women welcomed positive nutritional messages, which is consistent with other research [12,19]. 

One of the study limitations is the short time (2 weeks) between the participants receiving the resources and the follow up survey. It is possible that a longer time period may have lead to different knowledge and useability outcomes of the resources, perhaps providing stronger support for the usefulness of such resources. Conversely, given more time, the women may have reported reverting back to their usual level of fish/DHA consumption. A further unmeasured confounder which may have influenced the results of the present study is that the first survey was self-administered, whereas the second survey was administered over the phone. Speaking to someone may have altered responses because women may have felt embarrassed or wanted to impress the researcher. This study cannot be considered representative of all pregnant women due to the small sample size and recruitment being limited to public hospitals and not other locations. Nevertheless, this study provides encouraging results regarding the usefulness of well designed nutrition education materials in increasing DHA consumption of pregnant women. These encouraging results indicate a lager study, with a control group and dietary intake measurements would be warranted to test the cause and effect such education materials have on consumption of DHA in pregnant women in Australia. 

## 5. Conclusions

In summary, this study found that a pamphlet and shopping card expanded knowledge of DHA food sources, provided information on the benefits of DHA, and offered practical dietary suggestions for women during pregnancy. Pregnant women liked the design of the materials and found the information useful. Considering the lack of a control group, a recognized limitation of this study, the findings cannot conclude whether the pamphlet and shopping card would assist in increasing DHA consumption during pregnancy. However, due to the reported changes in behaviour and the increased awareness of DHA food sources, provision of such materials to pregnant women across Australia would be predicted to have positive outcomes for fetal development and maternal DHA stores [7] and further studies are warranted to determine whether this is in fact the case. The DHA pregnancy education pamphlet and card were useful nutrition education tools for the target group at both secondary and tertiary levels of education. A desire for these materials exists and should be addressed through availability of the pamphlet and card to all women during pregnancy. Randomised controlled trials with an appropriate control group and which involve methods, such as food frequency questionnaires before and after delivery of the materials, are warranted to demonstrate increased consumption of DHA as a direct result of these materials.
